# Awareness of the Scope of Oral and Maxillofacial Surgery and Referral Practices Among Medical Professionals in Telangana: A Cross-Sectional Study

**DOI:** 10.7759/cureus.111642

**Published:** 2026-06-28

**Authors:** Aziz Mohammed Sarah, Devarakonda Visalakshi, Vemulapally Santosh Krishna, B. Sridhar Reddy, Anusha Reddy, Aishwarya Ravikumar, Pallavi Narra, Rupa Rani Yerra, Vemuganti Supraja, Ajmera Prem Sagar

**Affiliations:** 1 Oral and Maxillofacial Surgery, Government Dental College and Hospital, Hyderabad, IND; 2 Public Health Dentistry, Sri Sai College of Dental Surgery, Hyderabad, IND

**Keywords:** barriers, interdisciplinary collaboration, perception of omfs, questionnaire, scope

## Abstract

Background: Oral and maxillofacial surgery (OMFS) is a specialized field that treats diseases, injuries, and defects in the head, neck, face, jaws, and oral tissues. Although its scope is broad, it is often not clearly or fully delineated within the medical community, thereby obscuring the specialty’s diagnostic and therapeutic reach and contributing to delayed or inappropriate referrals to other specialties, ultimately compromising patient care.

Objective: To assess awareness of the scope of OMFS among medical professionals in Telangana, evaluate referral patterns and referral paradigms, and explore measures to strengthen referral practices.

Methodology: A cross-sectional descriptive study was conducted using a structured questionnaire distributed through Google Forms among medical students and medical professionals studying or practicing in Telangana. The questionnaire assessed professional background, awareness of OMFS, referral behavior, perception of the specialty, collaboration with OMFS, and opinions on improving awareness. Data were analyzed descriptively using frequencies and percentages.

Results: A total of 342 participants were included. While 288 (84.2%) were aware of OMFS as a specialty, confidence in understanding its full scope was predominantly low to moderate. Referral to OMFS was frequently indirect, and many participants preferred other specialties, particularly Plastic surgery for facial trauma. Limited multidisciplinary integration and unclear referral pathways were identified as major barriers.

Conclusions: Although general awareness of OMFS is relatively high, understanding of its full scope and clinical role remains limited. Greater interdisciplinary exposure, structured referral pathways, and collaborative educational initiatives are required to improve recognition and utilization of the specialty.

## Introduction

Oral and maxillofacial surgery (OMFS) is a specialized branch of dentistry concerned with the diagnosis, surgical management, and treatment of diseases, injuries, and defects involving the oral cavity, jaws, face, and related structures. Its scope includes trauma care, reconstructive surgery, oral pathology, craniofacial deformities, salivary gland disease, dental implants, temporomandibular joint (TMJ) disorders, facial aesthetics, and head and neck pathology [1,2].

In routine clinical practice, patients with maxillofacial conditions are often first evaluated by emergency physicians, general surgeons, otorhinolaryngologists, plastic surgeons, ophthalmologists, or primary care physicians. When these professionals are not fully aware of the scope of OMFS, referrals may be delayed or misdirected, affecting treatment efficiency and continuity of care. Referral patterns for facial trauma also vary across institutions and specialties, highlighting the need for clearer interdisciplinary pathways [3].

Previous studies have shown that awareness and perception of OMFS among health care professionals vary widely. Many recognize the specialty by name but lack clarity regarding its clinical scope and referral role. Studies from India and other regions have consistently highlighted the need for improved understanding of OMFS among medical practitioners and students [1,4,5].

 A regional study from the same region by Vadepally et al. [6] reported limited perception of OMFS among both medical professionals and the general public. Similar findings were reported by Kunusoth et al. [7], where awareness existed, but confidence in the broader clinical scope remains limited.

Improving awareness is therefore essential to ensure timely referrals, effective inter-disciplinary collaborations, and optimal patient care. The primary objectives of this study were to assess the knowledge and awareness of OMFS among medical professionals in Telangana and to evaluate their referral practices for conditions within the scope of OMFS. The secondary objectives were to assess perceptions regarding the role and scope of OMFS in patient care, explore perceived barriers to appropriate referrals and effective interdisciplinary collaboration, and identify measures that may enhance awareness of OMFS and strengthen referral pathways and interdisciplinary engagement with the specialty.

## Materials and methods

Study design 

A cross-sectional, descriptive, questionnaire-based study was conducted to assess the awareness, perception, and referral preferences regarding OMFS among medical students and medical professionals in Telangana, India. The survey was conducted between November 2025 and April 2026, during which eligible participants were invited to complete the questionnaire through an online Google Forms platform. The survey questionnaire used in this study is provided in the Appendix.

Study setting and study population

The study was conducted through the Department of OMFS, Government Dental College and Hospital, Afzalgunj, Hyderabad. The study included participants from various medical colleges, teaching hospitals, government, and private healthcare facilities across Telangana. Participants included medical students at all levels, general practitioners, emergency medicine physicians, otorhinolaryngologists (ENT surgeons), plastic surgeons, general surgeons, anesthesiologists, ophthalmologists, oncologists, and other medical specialists practicing or undergoing medical training in Telangana.

Inclusion criteria

The study included medical students and medical professionals who voluntarily consented to participate in the survey. Participants were required to be either pursuing medical education or practicing medicine in Telangana. Individuals from all levels of medical training and professional experience were included, provided they met the study requirements and completed the questionnaire.

Exclusion criteria

Individuals who declined to provide informed consent or were unwilling to participate in the study were excluded. Furthermore, questionnaires with incomplete responses, missing essential data, or insufficient information for analysis were not included to ensure the accuracy of the study findings. Professionals from other healthcare disciplines, including dental professionals, homeopathic doctors, and alternative medical professionals, were excluded from the study.

Sample size

The minimum required sample size was calculated using the formula:

$n = \frac{Z^{2}\sigma^{2}}{d^{2}}$

where *Z* = 1.96, corresponding to a 95% confidence level, *σ *= 0.45 (based on previous studies) [4], and *d *= 0.05 (margin of error).

Based on this calculation, the minimum required sample size was 312 participants. To enhance statistical power and representativeness, data collection was extended beyond this threshold, yielding a final sample of 342 complete responses for analysis.

Study tool

Data were collected using a structured questionnaire designed to evaluate awareness and perception of OMFS. It consisted of five sections: 

Section 1: Electronic informed consent

Section 2: Professional profile

This section collected professional demographic data, including participant designation, district of study or practice, and workplace setting. To ensure absolute participant anonymity and data privacy, no identifiable personal demographic data was collected.

Section 3: Awareness and perception of OMFS

This section assessed participants' awareness of OMFS as a distinct surgical specialty, previous interactions with OMFS, perceptions regarding the scope of practice of OMFS surgeons, and confidence in identifying procedures commonly performed by OMFS.

Section 4: Specialty preference for clinical conditions

Participants were asked to identify the specialty they considered most appropriate for managing various clinical conditions involving the oral and maxillofacial region. Conditions include facial fractures, salivary gland disorders, orofacial malignancies, congenital and acquired craniofacial deformities, craniofacial syndromes, deep neck infections, and other maxillofacial pathologies.

Section: 5 Referral patterns, barriers to referral, and improvement strategies

This section evaluated participants' collaboration with OMFS, referral patterns, perceived barriers to referral to OMFS, and suggestions for improving the same.

Validity

The questionnaire was developed as a hybrid instrument. Core items assessing baseline awareness of OMFS and referral practices were adapted from previously published studies. Additional items exploring barriers to interprofessional collaboration and educational opportunities were developed by the authors to address distinct regional healthcare gaps. Face and content validity were established through review by a panel consisting of three senior oral and maxillofacial surgeons and two public health dentists. The questionnaire was subsequently reviewed and revised according to their recommendations to ensure clarity, appropriate clinical terminology, and adequate coverage of the study objectives. Internal consistency of the awareness scale was evaluated using Cronbach’s alpha and was found to be good, with *α* = 0.84.

Pilot testing

A pilot study was conducted among 15 medical professionals who met the study inclusion criteria. The pilot aimed to assess the clarity and comprehensibility of the questionnaire, identify any ambiguities, and evaluate the average completion time. Based on participant feedback, the questionnaire was found to be clear and easy to understand, with no major modifications required. Responses obtained during the pilot phase were excluded from the final analysis to prevent sample contamination.

Reliability

As the questionnaire consists of independent categorical and nominal items assessing distinct clinical and institutional scenarios rather than a unified psychometric trait, statistical internal consistency testing was not methodologically indicated. Instrument reliability was secured procedurally by routing the newly framed items through expert panel calibration and using strictly closed-ended, multiple-choice options across all domains. These measures helped ensure stability, consistency in response options, and uniform data collection across the study population.

Data collection

The questionnaire was converted into an electronic format using Google Forms and distributed through professional networks, institutional communication channels, and social media platforms. Participation was voluntary, and informed consent was obtained electronically before the commencement of the survey. Responses were collected anonymously, and no personally identifiable information was recorded.

Statistical analysis

Data were summarized using descriptive statistics and presented as frequencies and percentages. Statistical analyses were performed on the combined cohort of practicing clinicians and undergraduate medical students using IBM SPSS Statistics for Windows, Version 20.0 (IBM Corp., Armonk, NY). Accordingly, all results presented in the study reflect aggregate data from the overall cohort rather than separate subgroup analyses.

Ethical considerations

Participation was voluntary, and informed consent was obtained electronically before questionnaire completion. Responses were collected anonymously, and no personally identifiable information was obtained. The survey assessed professional awareness and referral practices and did not involve patient data or clinical interventions.

## Results

A total of 342 participants were included, of whom 256 (74.85%) were practicing medical clinicians and 86 (25.15%) were undergraduate medical students. Statistical analyses were performed on an aggregate basis to provide a comprehensive baseline assessment of OMFS awareness across the institutional hierarchy. Because the student subgroup of 86 (25.15%) is not yet independently involved in active patient referral processes, their responses in the referral domains reflect theoretical knowledge and perceived institutional pathways, whereas active clinical referral behavior is driven by the practicing clinician cohort. The distribution of study participants according to their primary professional role and clinical practice setting is presented in Table 1. 

**Table 1:** Socio-professional demographic characteristics of the study participants. Traditional personal identifiers (such as age and gender) were omitted from the questionnaire to guarantee strict participant anonymity in an online, voluntary survey environment.

Demographic variable	Participant category	Frequency	Percentage
Current primary professional role	Medical Student (MBBS/MD/DO)	86	25.15%
	General Practitioner/Family Medicine Physician	28	8.19%
	Emergency Medicine Physician (Trainee/Consultant)	19	5.56%
	ENT/Otolaryngology Surgeon (Trainee/Consultant)	21	6.14%
	Plastic and Reconstructive Surgeon (Trainee/Consultant)	29	8.48%
	General Surgeon (Trainee/Consultant)	37	10.82%
	Anesthesiologist	27	7.89%
	Ophthalmologist	12	3.51%
	Oncologist	7	2.05%
	Other	76	22.22%
Primary practice setting	District General Hospital/Community Hospital	111	32.46%
	Large Academic/Tertiary Care Hospital	98	28.65%
	Private Clinic/Office	61	17.84%
	Academic Institution (Non-clinical role)	40	11.70%
	Others	32	9.36%
Geographical distribution	Telangana	342	100%

Awareness of OMFS as a specialty was 288 (84.2%); however, respondents' confidence in understanding the full scope of the specialty, rated on a scale from 1 (not at all confident) to 5 (very confident), was predominantly low to moderate, with 107 (31.3%) selecting a score of 2 and 108 (31.6%) selecting a score of 3.

The frequency of participant interactions with an OMFS service or department within their primary workplace is outlined in Table 2.

**Table 2:** Frequency of interaction with OMFS. OMFS, oral and maxillofacial surgery

Interaction frequency	Number of responses	Percentage
Rarely	133	38.89%
Never	73	21.35%
Monthly	66	19.30%
Weekly	46	13.445%
Daily	24	7.01%
Total	342	100%

Referral patterns to OMFS

The distribution of referral practices among participants for patients requiring OMFS consultation is given in Table 3.

**Table 3:** Referral patterns to OMFS. OMFS, oral and maxillofacial surgery

Referral method	Number of responses	Percentage
No referral	146	42.69%
Secondary referral	112	32.75%
Direct referral	84	24.56%
Total	342	100%

The general perceptions of OMFS among the participants are shown in Table 4.

**Table 4:** Perception of OMFS professionals. OMFS, oral and maxillofacial surgery

Perception statement	Response count	Percentage
Unsure of exact professional alignment (medical vs. dental)	121	35.38%
Primarily, dentists who perform minor extractions	81	23.68%
Perform minor oral surgery procedures like surgical tooth extractions, wisdom tooth removal, apical surgeries, biopsies, and dental implants, but not very extensive surgeries	76	22.22%
Equivalent to a general surgeon for the lower face only	37	10.82%
Highly specialized surgeons who manage complex head/neck conditions alongside other specialties	27	7.89%
Total	342	100%

Table 5 summarizes participants' specialty preferences for the management of specific clinical conditions.

**Table 5:** Preferred specialty for each clinical scenario. Note: Respondents could select more than one option; percentages are calculated based on the total number of respondents and may sum to more than 100. OMFS, oral and maxillofacial surgery

Clinical condition or procedure	Plastic surgery, *n* (%)	ENT, *n* (%)	General surgery or trauma service, *n* (%)	OMFS, *n* (%)	Oncology, *n* (%)	Ophthalmology or oculoplastic surgery, *n* (%)	Whichever service is available on call, *n* (%)
Acute facial trauma	212 (61.98%)	75 (21.93%)	110 (32.2%)	119 (34.8%)	-		73 (21.4%)
Complex maxillomandibular fracture	162 (47.37%)	54 (15.8%)	58 (17%)	219 (64.035%)			
Nasal bone and orbital fractures	203 (59.4%)	198 (57.8%)	39 (11.5%)	93 (27.3%)		189 (55.2%)	
Orofacial malignancy	107 (31.29%)	44 (12.87%)	49 (14.3%)	65 (19.01%)	246 (72%)		
Salivary gland pathology	120 (35.09%)	183 (53.5%)	46 (13.45%)	110 (32.16%)	128 (37.43%)		
Reconstruction or correction of acquired facial defects (maxillo-mandibular/nasal/frontal/malar defects)	240 (70.2%)	39 (11.4%)	40 (13.2%)	82 (23.98%)			
Correction of congenital deformities of the face (lower or midface proclination or retroclination) or oral cavity (cleft lip and cleft palate)	234 (68.4%)	42 (12.3%)	73 (21.3%)	115 (33.63%)			
Correction of craniofacial deformities (e.g., Parry-Romberg syndrome, Crouzon syndrome, etc.)	223 (65.2%)	48 (14.04%)	42 (12.2%)	76 (22.22%)			
Facial and deep neck space infections (including Ludwig's angina)	65 (19.1%)	186 (54.3%)	118 (34.6%)	122 (35.8%)			
Management of obstructive sleep apnea	91 (26.7%)	219 (64%)	64 (18.6%)	74 (21.6%)			

In cases of atypical facial pain, respondents most frequently selected a neurologist or pain physician (245, 71.6%), followed by OMFS (94, 27.5%), plastic and reconstructive surgery (50, 14.62%), and ENT/otolaryngology (47, 13.74%).

The reported frequency of OMFS inclusion in multidisciplinary team (MDT) meetings for relevant conditions, such as Head and Neck Cancer MDTs and Trauma MDTs, is presented in Table 6.

**Table 6:** Inclusion of OMFS in multidisciplinary team (MDT) meetings. OMFS, oral and maxillofacial surgery

Response	n	Percentage
Occasionally included	128	37.43%
Rarely/Never	89	26.02%
Not applicable to their current role or setting.	79	23.10%
Always included	46	13.45%
Total	342	100%

Most respondents agreed that unclear boundaries between OMFS, ENT, and Plastic Surgery may lead to inefficiencies and confusion in patient care pathways, with 157 (45.9%) agreeing and 42 (12.3%) strongly agreeing. Neutral responses accounted for 98 (28.65%), while 30 (8.77%) disagreed and 15 (4.4%) strongly disagreed.

Barriers to effective referrals

The perceived barriers to effective collaboration with OMFS are listed in Table 7.

**Table 7:** Barriers to effective referrals. Note: This was a multiple-choice question in which respondents could select more than one barrier; therefore, the total response count and percentages exceed 342 and 100%, respectively. OFMS, oral and maxillofacial surgery; MDT, multidisciplinary team

Collaboration barrier	n	Percentage
No collaboration due to the lack of OMFS in our institution	155	45.32%
Lack of clarity on referral protocols	116	33.92%
Limited opportunities for joint training/MDT meetings	91	26.61%
Perception of *turf wars* or competition for cases	83	24.27%
Physical separation of departments (e.g., different buildings)	52	15.20%
No significant barriers exist	42	12.28%

Strategies to improve awareness

The participants' responses regarding strategies to enhance collaboration with OMFS are displayed in Table 8.

**Table 8:** Strategies to improve awareness. Note: This was a multiple-choice question in which respondents could select more than one strategy; therefore, the total response count and percentages exceed 342 and 100%, respectively.

Barrier	Response count	Percentage
Increased joint specialty meetings and educational seminars	177	51.75%
More collaborative research opportunities	143	41.81%
Clearer, written guidelines on hospital referral pathways	125	36.55%
Mandatory OMFS rotations during medical school/residency	92	26.9%
Changing the name of the specialty to sound more *medical*	60	17.54%

Most respondents reported that they did not know enough to comment on the scope overlap between OMFS, ENT, and Plastic Surgery (156, 45.5%). Among the remaining respondents, 111 (32.5%) indicated that there was significant overlap confusing referrals; 38 (11.1%) believed that overlap existed only in minor areas while the main scopes remained distinct, and 37 (10.8%) reported minimal overlap, with roles being very clearly defined.

Study the impact on awareness

Most respondents (250, 73.1%) acknowledged that the study helped enhance their awareness and knowledge of OMFS, as depicted in Table 9.

**Table 9:** Study impact on awareness.

Response	n	Percentage
Yes	250	73.1
No	28	8.1
I am not sure	64	18.8

The final open-ended section of the questionnaire provided additional insights into participants' perceptions and experiences regarding OMFS. Although these responses were not subjected to formal qualitative analysis, several recurring themes were noted.

Many respondents emphasized the need for greater awareness of OMFS and its scope of practice among both medical and dental professionals. Participants from institutions with dental departments reported being aware of the existence of OMFS; however, this awareness was often limited and largely attributed to informal interactions rather than structured educational exposure (e.g., "We don't have a specialized OMFS branch, but through casual interactions with the dental department, we meet oral and maxillofacial surgeons; it is solely because of them that I am aware of this specialty").

Conversely, respondents from institutions without OMFS services frequently reported limited exposure to the specialty and uncertainty regarding its clinical scope. Several participants indicated that, in the absence of a dedicated OMFS department, relevant cases were commonly referred to other specialties such as Plastic Surgery, Surgical Oncology, or ENT (e.g., "Due to the lack of availability of an oro-maxillary dept, doctors are referring cases to other specialists like plastic, oncology, or ENT").

Common suggestions for improving awareness included increased interdepartmental interaction through panel discussions, case discussions, collaborative research, and multidisciplinary meetings, as well as compulsory hospital postings or rotations to improve visibility of the specialty. Several respondents also highlighted the importance of clearer referral pathways, stronger OMFS representation in hospitals and tertiary care centers, and greater recruitment or establishment of OMFS departments. A smaller number of comments supported public awareness initiatives, undergraduate-level integration of teaching, and more clinically based talks to improve understanding of the specialty's role.

## Discussion

OMFS in India has undergone substantial evolution since its emergence in the 1950s, progressing from a discipline primarily focused on dentoalveolar surgery and minor trauma to a comprehensive surgical specialty managing complex maxillofacial trauma, head and neck pathologies, orthognathic deformities, and facial reconstructive and aesthetic procedures. Formal training programs were established by the mid-1970s, and over the past five to six decades, the specialty has expanded considerably in both scope and capability. However, despite more than half a century of advancement, public and professional perception of its scope of practice remains below par. The specialty is frequently confused with other surgical specialties within both dentistry and medicine, with which it shares anatomical regions or clinical scope [8]. OMFS continues to be widely perceived in its earlier, restricted role, often associated only with routine dentoalveolar procedures, resulting in inadequate awareness and suboptimal referral patterns among medical professionals. This disparity between the contemporary scope of OMFS and its prevailing perception highlights the need to improve awareness and optimize referral pathways.

This study was conducted to assess awareness of the scope of OMFS among medical professionals in Telangana, evaluate existing referral patterns to the specialty, and identify strategies to improve both awareness and referral practices. The study demonstrates that while OMFS is generally recognized as a specialty among medical professionals, an understanding of its full scope remains limited. In reality, awareness of a specialty’s existence alone does not necessarily translate into appropriate referral behavior or effective interdisciplinary collaboration [1,9].

Low to moderate confidence in identifying OMFS procedures was directly reflected in referral patterns, where secondary consultation or no consultation was significantly more common than direct referral. This suggests that OMFS is not consistently considered the primary specialty for maxillofacial conditions [10,11]. Similar trends have been observed in referral studies, where specialty choice is heavily influenced by institutional familiarity and established practices rather than objective clinical scope [3,12].

When evaluating specific regional data, studies by Vadepally et al. [6] and Kunusoth et al. [7], conducted in the same region as the present study, reported comparable findings of limited awareness and unclear perception of the specialty. In comparison, the present study indicates a modest improvement in general awareness; however, gaps in referral patterns and in the depth of understanding of the scope persist.

This narrow perception of OMFS scope is a global issue rather than an isolated regional phenomenon. Many of our respondents associated the specialty primarily with dentistry or minor oral surgical procedures, while only a few recognized its role in managing complex surgical conditions. Similar findings have been reported by Ameerally et al. [13] and Hunter et al. [14] in the United Kingdom. In line with this, Lau’s 2014 study [15] reveals that a significant majority (54%) of those familiar with the term OMFS were unaware of the actual scope of OMFS services, highlighting substantial overlap and baseline lack of awareness. Collectively, these regional and international findings indicate that limited recognition of the breadth of OMFS practice remains a persistent challenge across different healthcare systems.

The preference for plastic surgery in managing facial trauma and correction of congenital and acquired facial defects (both hard and soft tissues) highlights a significant gap in awareness. Although plastic surgery contributes to facial reconstruction, OMFS plays a central role in the management of injuries involving the jaws, facial skeleton, and oral structures [3,16,17].

ENT surgeons were more frequently preferred for conditions such as nasal bone fractures, benign salivary gland pathologies, obstructive sleep apnea, and deep neck space infections. Furthermore, neurophysicians and general physicians were often favored over OMFS in the management of atypical facial pain, including conditions such as trigeminal neuralgia and myofascial pain dysfunction syndrome, and oncologists for malignancies involving the maxillofacial region.

Overall, these referral preferences indicate that the scope of OMFS remains poorly understood in areas where the clinical responsibilities overlap with other specialties. This finding is consistent with the observations of Adewole and Akinwande [18], who reported that OMFS is frequently viewed through a restricted clinical perspective despite its broad involvement in the management of maxillofacial conditions. Such gaps in understanding the scope may hinder optimal referral practices, signifying the need for targeted awareness initiatives among healthcare professionals. In this context, Rocha et al. [19] emphasized that the specialty must actively broaden its horizons by expanding its interdisciplinary presence and clinical footprint.

The study revealed that the involvement of OMFS in multidisciplinary discussions was inconsistent. Key barriers identified included the absence of dedicated OMFS departments and the lack of clearly defined referral pathways. Participants suggested that joint academic activities such as seminars, interdisciplinary meetings, and collaborative research could improve awareness and strengthen interdisciplinary engagement.

An important observation is that only a limited number of hospitals or medical institutions, predominantly in the private sector, have well-established OMFS departments, while such structured departments are less commonly available in many government hospitals. Consequently, medical students and trainees in these settings may have limited exposure to OMFS during their training, leading to reduced familiarity with the specialty.

A clear difference was noted between institutions associated with dental colleges and those without exposure to OMFS. In institutions with dental colleges, medical professionals have some familiarity with OMFS and are more likely to refer relevant cases to the specialty, although referrals were not fully comprehensive, and other departments were still often preferred in certain situations. This suggests that referral to OMFS is strongly influenced by the institutional environment and academic exposure, which generally contribute to continued referral of relevant cases to other departments even after completion of education, reflecting patterns developed during their formative years. Comparable findings were reported by Mohammad Kamal et al. [20], who emphasized the need to revisit medical and dental curricula to enhance educational content related to OMFS procedures, along with increasing clinical awareness of the specialty among healthcare professionals and institutions. Collectively, these observations reinforce the importance of adequate academic exposure and the establishment of fully operational OMFS departments across teaching and clinical institutions, including government and private sectors, to ensure appropriate training, clearer referral pathways, and optimal patient care.

Despite increasing academic research in this area, improvements in referral patterns and comprehensive awareness have been gradual. Nevertheless, there appears to be a positive trend toward increasing recognition of the specialty, with more institutions beginning to acknowledge its scope and incorporate OMFS into multidisciplinary care and case discussions.

In some settings where OMFS departments are present, overlapping scopes of practice and unclear delineation of responsibilities between departments may also influence referral decisions, highlighting the need for clearer role definition and more collaborative referral pathways.

A coordinated and collaborative approach across all specialties is essential to ensure appropriate integration of OMFS into patient management pathways. Strengthening interdepartmental interaction, clearly delineating the scope of practice, and fostering an environment of shared learning can contribute to improved patient outcomes and broader recognition of the specialty.

The barriers identified in this study were mainly structural, including the absence of OMFS departments and unclear referral pathways, indicating that awareness alone is insufficient without institutional support [8,11].

Greater involvement of OMFS surgeons in interdisciplinary medical conferences may further enhance awareness of the specialty among medical professionals. As suggested by Ifeacho et al. [21], effective communication of the scope and clinical responsibilities of OMFS to both healthcare professionals and the general public remains essential for facilitating appropriate referral pathways. In this regard, keynote lectures, case presentations, panel discussions, joint meetings, seminars, collaborative research, and participation in multidisciplinary case discussions can collectively broaden understanding of the scope, relevance, and clinical contributions of OMFS. Such academic engagement may strengthen interdepartmental collaboration, improve appreciation of OMFS input in patient management, and facilitate more appropriate referral to the specialty [5,7].

Limitations: As a cross-sectional survey, it reflects participants' knowledge, awareness, perceptions, and referral practices at a single point in time and does not permit causal inferences. The study was conducted among medical professionals and medical students in Telangana; therefore, the findings may not be generalizable to other regions. Participation was voluntary, introducing the potential for selection bias, while the absence of institutional affiliation and response rate data limited assessment of institutional representation and non-response bias. Furthermore, as the findings were based on self-reported questionnaire responses, they may have been influenced by recall bias and variations in the interpretation of survey items.

Nevertheless, the study provides valuable insights into awareness, perceptions, and referral patterns related to OMFS among medical professionals in Telangana. Including participants from multiple medical specialties and levels of training, it offers a broad overview of current interdisciplinary understanding of the specialty and identifies areas where awareness, referral patterns, and professional collaboration may be strengthened. The findings may serve as a foundation for future educational initiatives and larger multi-center studies aimed at optimizing the integration of OMFS within multidisciplinary patient care.

Notably, 73.1% of participants reported that the questionnaire enhanced their understanding of the specialty, highlighting the potential value of educational initiatives in improving understanding of the role and scope of OMFS. 

The study suggests that despite the increasing recognition of OMFS among medical professionals, important gaps remain in the understanding of its full scope. Strengthening institutional presence, promoting multidisciplinary engagement, and implementing structured awareness initiatives may further enhance appropriate utilization of the specialty and improve patient care pathways.

## Conclusions

Although the awareness of OMFS among medical professionals in Telangana was generally high, important gaps remain in the understanding of its full scope and referral role. The continued preference for alternative specialties in several areas of overlapping clinical responsibility suggests that awareness alone may not be sufficient to ensure appropriate utilization of OMFS services. Greater educational exposure, clearer referral pathways, and stronger interdisciplinary engagement may help improve recognition of the specialty and facilitate more appropriate referral practices. These findings provide valuable baseline data to guide future educational and institutional initiatives aimed at strengthening the role of OMFS within multidisciplinary patient care.

## Appendices

Appendix 

**Table 10:** Study Questionnaire

Sl no.	Survey Question / Item Title	Response options or formats
Section 1	Study Information & Electronic Informed Consent	
Q1	Informed Consent: I have read the purpose of this survey, understand that my data is fully anonymized, and I agree to participate.	Yes No
Section 2	Demographic and Professional Profile	
Q2	Current Primary Profession/Role:	Medical Student (MBBS/MD/DO) General Practitioner/Family Medicine Physician Emergency Medicine Physician ENT/Otolaryngology Surgeon Plastic & Reconstructive Surgeon General Surgeon Anesthesiologist Ophthalmologist Oncologist Other (Please specify)
Q3	Which district in Telangana do you practice or study?	Open-ended text / Regional dropdown selection
Q4	Primary Practice Setting:	Large Academic/Tertiary Care Hospital District General Hospital/Community Hospital Private Clinic/Office Academic Institution (Non-clinical role) Others (Please specify)
Section 3	Baseline Awareness & Inter-specialty Interaction	
Q5	Are you aware of oral and maxillofacial surgery as a distinct specialty in the field of medicine?	Yes No Maybe
Q6	In your primary workplace, how often do you interact with an OMFS service/department?	Daily Weekly Monthly Rarely Never
Q7	On a scale of 1 (Very Unconfident) to 5 (Very Confident), how confident are you in identifying the full scope of procedures performed by an OMFS specialist?	Linear scale: 1 (Very unconfident) to 5 (Fully confident)
Q8	How do you typically refer a patient to OMFS?	Direct consultation/referral to the on-call service/clinic Indirect / Secondary referral Via a different specialty first (e.g., ENT or Plastics) No referral
Q9	Which of these statements best describes your general perception of OMFS professionals?	Primarily dentists who perform minor extractions. Highly specialized surgeons managing complex head/neck conditions. Equivalent to a general surgeon for the lower face only. Unsure of exact professional alignment (medical vs. dental). Perform minor oral surgery procedures like surgical tooth extractions, wisdom tooth removal, apical surgeries, biopsies, and dental implants but not very extensive surgeries
Section 4	Specialty Preferences & Clinical Scenarios	(Select ALL qualified specialties per row)
Q10	Acute facial trauma (e.g., cheekbone, nasal, maxillary, or mandibular fracture)	Plastic Surgery | ENT | OMFS | Gen Surgery/Trauma | Orthopedics | Depends on "on call"
Q11	Management of complex maxillomandibular fracture	ENT/Otolaryngology | Plastic Surgery | General Surgery | OMFS
Q12	Nasal bone and orbital fractures	ENT/Otolaryngology | Plastic Surgery | General Surgery | OMFS | Ophthalmology/Oculoplastic
Q13	Orofacial malignancy (Carcinoma buccal mucosa, palate, tongue, mandible, maxilla, facial ulcers, bone tumors)	ENT/Otolaryngology | Plastic Surgery | General Surgery | OMFS | Oncologist
Q14	Salivary gland pathology (both benign and malignant)	ENT/Otolaryngology | Plastic Surgery | General Surgery | OMFS | Oncologist
Q15	Reconstruction or correction of acquired facial defects (maxillo-mandibular/nasal/frontal/malar defects)	Plastic Surgery | General Surgery | ENT/Otolaryngology | OMFS
Q16	Correction of congenital deformities of face (lower/midface proclination/retroclination) or oral cavity (cleft lip/palate)	Plastic Surgery | General Surgery | ENT/Otolaryngology | OMFS
Q17	Correction of craniofacial deformities (e.g., Parry Rhomberg syndrome, Crouzon syndrome)	Plastic Surgery | General Surgery | ENT/Otolaryngology | OMFS
Q18	Atypical facial pain (e.g., Trigeminal neuralgia, Myofascial pain dysfunction syndrome)	Plastic Surgery | Neuro Physician | ENT/Otolaryngology | OMFS | General Physician
Q19	Facial and deep neck space infections (including Ludwig's angina)	Plastic Surgery | ENT/Otolaryngology | General Surgery | OMFS
Q20	Management of Obstructive sleep apnea	ENT/Otolaryngology | Plastic Surgery | General Surgery | OMFS
Section 5	Barriers, Collaboration, & Educational Opportunities	
Q21	How often is the OMFS service included in multidisciplinary team (MDT) meetings for relevant conditions?	Always included and actively participate Included occasionally, but not a primary member Rarely or never included Not applicable to my current role/setting
Q22	Do you believe that unclear boundaries between OMFS, ENT, and Plastic Surgery lead to inefficiencies and confusion?	Strongly agree Agree Neutral Disagree Strongly disagree
Q23	Which of the following is the biggest barrier to effective collaboration between your specialty and OMFS at your institution?	Lack of clarity on referral protocols Physical separation of departments (different buildings) Perception of "turf wars" or competition for cases Limited opportunities for joint training/MDT meetings No significant barriers exist No collaboration due to lack of OMFS in our institution
Q24	What methods do you believe would be most effective in improving awareness of the OMFS scope of practice?	Mandatory OMFS rotations during medical school/residency Increased joint specialty meetings and educational seminars Clearer, written guidelines on hospital referral pathways More collaborative research opportunities Changing the name of the specialty to sound more "medical"
Q25	How interested would you be in attending a continuing medical education (CME) workshop focused specifically on OMFS?	Very interested Interested Neutral Not interested
Q26	Which phrase best describes your understanding of the scope creep/overlap between OMFS, ENT, and Plastic Surgery?	Significant overlap, causing confusion for referrals Overlap exists in minor areas, but main scopes are distinct Minimal overlap, roles are very clearly defined Don't know enough to comment
Q27	Therefore, was this study helpful in enhancing your knowledge and awareness regarding OMFS?	Yes No, I am not sure
Q28	Please provide any additional remarks or suggestions regarding OMFS referral pathways or collaboration.	Open-ended text field
